# Diabetes Mellitus Contributes to Idiopathic Pulmonary Fibrosis: A Review From Clinical Appearance to Possible Pathogenesis

**DOI:** 10.3389/fpubh.2020.00196

**Published:** 2020-06-03

**Authors:** Dongguang Wang, Yao Ma, Xiang Tong, Yonggang Zhang, Hong Fan

**Affiliations:** ^1^Department of Respiratory and Critical Care Medicine, West China Hospital, West China School of Medicine, Sichuan University, Chengdu, China; ^2^The Center of Gerontology and Geriatrics, West China Hospital, West China School of Medicine, Sichuan University, Chengdu, China; ^3^Department of Periodical Press, West China Hospital, Sichuan University, Chengdu, China

**Keywords:** diabetic pulmonary fibrosis, diabetic complications, diabetes mellitus, idiopathic pulmonary fibrosis, risk factors, potential mechanisms

## Abstract

Diabetes mellitus is a systematic metabolic disease characterized by persistent hyperglycemia, which complications often involve multiple organs and systems including vessels, kidneys, retinas, and nervous system. Idiopathic pulmonary fibrosis is a chronic, progressive, fibrotic disease with usual interstitial pneumonia patterns. With in-depth research, diabetic related lung injury has been confirmed, and the lung is also considered as one of the targeted organs of diabetes, which mainly manifests as the pulmonary fibrosis. Based on that, this review discusses the association between diabetes mellitus and idiopathic pulmonary fibrosis from clinical findings to possible mechanisms.

## Introduction

Idiopathic pulmonary fibrosis (IPF) is defined as a chronic, progressive, fibrosing interstitial lung disease with the histologic appearance of usual interstitial pneumonia (UIP) (1). The conservative estimate of incidence on IPF is 3~9 cases per 100,000 people per year for Europe and North America (2). The prognosis for patients with IPF is quite poor, with a median survival of 3~5 years from the initial diagnosis if not treated (1). However, the etiology remains little understood.

Diabetes mellitus (DM) is a systemic, metabolic disorder characterized by insulin deficiency or resistance, chronic hyperglycemia, and micro- and macro-vascular bed damage. In the long run, diabetes deeply affects multiple organs throughout the body, especially kidney, retina, heart, and brain, making loss of function of these crucially important organs and bringing about death (3–6). The lung consists of abundant alveolar-capillary network and connective tissue, suggesting that it may be targeted by diabetic micro-vascular damage. Unfortunately, the correlation has often been disregarded and there is lack of research and evidence considering lung as a target for the diabetic impairment. In recent years, several studies have revealed that hyperglycemia could lead to interstitial fibrotic changes and alveolar microangiopathy (7–10). Here, we review the present clinical and experimental evidence, aiming to explore the role of diabetes on idiopathic pulmonary fibrosis (Table 1).

**Table 1:** Characteristics in diabetic pulmonary fibrosis patients.

**Epidemiology**	**Examinations**	**Potential treatment**
• Higher incidence of co-morbid disease • Longer hospital stay • More in-ward frequency • Higher mortality	• Spirometry: worse diffusion function and ventilation dysfunction • CT scan: more significant UIP pattern • Pathology: increasing thickness of basal lamina and more fibrosis	• Metformin • GLP-1 receptor agonists: liraglutide, exendin-4 • PPAR-γ agonists: rosiglitazone, ciglitazone

## Epidemiology

Since Schuyler et al. first demonstrated loss of pulmonary elastic recoil in the diabetics and proposed the abnormalities might be manifestations of extensive abnormal collagen and elastin depositions in the 1970's (11), more and more research has investigated the link between diabetes and idiopathic pulmonary fibrosis. Recent epidemiological research in populations with different genetic background are more prone to the opinion that diabetes is an independent risk factor for idiopathic pulmonary fibrosis, with the prevalence of IPF accompanied by DM estimated to be 10~42%, and the reported result is still consistent even if excluding the interference of patients treated with glucocorticoids (12–18). Following the poor prognosis in IPF patients, diabetes were reported to be a risk factor with an obviously higher mortality in this population (HR 2.5, 95%CI 1.04~5.9) (19). In addition, other studies showed a higher incidence of co-morbid diseases, longer hospital length of stay, and more frequent hospital admission and readmission in IPF patients with diabetes, which also indicated a worse prognosis (12, 15). However, the results above came from studies conducted in several countries with small populations, and amounts of cases included were restricted, more large-sample studies are needed for the overall prognosis assessment of diabetic pulmonary fibrosis in the near future.

## Clinical Characteristics

### Spirometry

Spirometry is regarded one of the most common measuring tools used to evaluate the severity of respiratory disease. Pulmonary function test on IPF patients mainly shows a ventilatory dysfunction and decreased diffusion capacity. Several longitudinal clinical studies have explored changes of pulmonary function in diabetic individuals. A prospective observational study from Europe found a visible decline of lung volumes and airflow limitation (decreased FVC, FEV_1_, VC, and PEF) in type 2 diabetes patients (20). Likewise, a 5-years prospective study in Japan showed that diabetes was associated with the restrictive pulmonary function impairment (multivariable adjusted HR 1.57, 95%CI 1.04~2.36) (21). What's more, the recent results from meta-analyses supported the epidemiological evidence as well, with one showing that diabetes free from overt pulmonary disease was associated with impaired pulmonary function in a restrictive pattern and the other finding a lower FVC than FEV_1_ and DL_CO_ in diabetic individuals (22, 23). While the reduction of spirometric parameters in diabetes seems to be consistent with that caused by IPF, inferences regarding casual effects cannot be made due to lack of evidence of pulmonary fibrosis from these diabetic populations. For this purpose, more longitudinal diabetic research is needed to evaluate the dynamic changes of pulmonary fibrosis indexes in the future.

### Imagings

Apart from spirometry, computed tomography (CT) examination is also essential for differential diagnosis of pulmonary lesions. Imaging in patients with IPF usually manifests as UIP pattern including reticular and honeycomb patterns. Kim et al. revealed that IPF patients with diabetes were more likely found the UIP pattern on high resolution computed tomography (HRCT) than those without diabetes (15). Similar results was also demonstrated from a Chinese study, in which more significant presence of funicular and reticular changes were found in diabetic participates compared to normal controls without type 1 diabetes (8). Unfortunately, there is still a lack of enough prospective data to confirm the imaging changes in diabetic patients.

### Pathological Changes

Histologically, the basal lamina (BL) is an extracellular scaffold located between parenchymal cells and connective tissue, by its presence compensating new cells normally apoptotic or damaged during the injury (24). If the integrity of basal lamina is destroyed, the repair in most organs results in pathologic fibrosis and loss of organ function. Over the past decades, several studies focused on diabetes had demonstrated a wide alterations of BL in different organs including lungs. Vracko et al. found a thickness of basal lamina both in epithelial and capillary of alveoli from diabetic individuals, and similar morphological changes were verified by Matsubara and other researchers in the 1990's (7, 25–27). Recently, experiments performed in induced diabetic murine models have also confirmed the clinical findings. Diabetic mice and rats were both found a significant thickening of alveolar septa and more fibrosis than non-diabetic controls (8, 28, 29). Since the collagen is the main component of basal lamina, increasing thickness of basal lamina may be the microscopic appearance of idiopathic pulmonary fibrosis (30), and as a result, the pathological changes mentioned above further strengthen the link between diabetes and idiopathic pulmonary fibrosis.

## Possible Pathogenesis

The exact pathogenesis of IPF has not been fully elucidated. Traditionally, the evolution of IPF could be divided into three stages: (1) initiation stage, oxidative stress damage from all causes is the initiation factor of IPF; (2) progression stage, inflammation of alveoli, activation of immune cells, and secretion of various pro-inflammatory factors, causing injuries of epithelial, endothelial, and interstitial cells, collagenous tissues, and basement membranes; (3) outcome stage, the formation of pulmonary fibrosis, characterized by proliferation of fibroblasts and myofibroblasts, deposition of extracellular matrix (ECM) and destruction of structure in the lung tissues, eventually leading to chronic respiratory failure. Epithelial-mesenchymal transition (EMT), with characteristics of the increase of fibrotic markers such as α-smooth muscle actin (α-SMA) and vimentin, and the reduction of epithelial markers such as E-cadherin, has been affirmed the crucial tache in pathological pulmonary fibrosis (31). Hyperglycemia promotes to the development of pulmonary fibrosis by participating in all these processes in diabetic animals.

### Advanced Glycation End-Products (AGEs) and the Receptor for Advanced Glycation End-Products (RAGEs)

The distribution of AGEs in wide range of organs is limited at a low basal level under normal conditions, while diabetes induces AGEs to accumulate in the lungs (32, 33). Using the streptozotocin (STZ) induced diabetic mice, researchers detected an extensive accumulation of AGEs and pathological hyperplasia of extracellular matrix and interstitial connective tissue in the lung (34, 35). RAGEs, a cell surface receptor for AGEs, have been detected as an antagonist for the clearance of AGE proteins. Cumulative evidence has confirmed a spontaneously pulmonary fibrosis-like alteration in RAGE (-/-) mice (36, 37). RAGEs blocked the activation of Smad2, ERK, and JNK signals, playing a negative role on transforming growth factor beta (TGF-β) induced EMT in A549 cells (38, 39). However, in contradiction to this conclusion, He et al. found a decreased expression of RAGEs and fibroblast lesions in RAGE (-/-)-mice (40), and overexpression of RAGEs increased the oxidative stress and mRNA expression of elastin both in fibroblasts and co-cultured lung epithelial cells (41). Thus, the need of further studies on the function of RAGEs in pulmonary fibrosis is clear now.

### Sirtuins (Sirt)

Sirt are class of NAD^+^ dependent proteins, mediating the process of secretion of insulin, cell cycle, and cell apoptosis. Shaikh reviewed the role of sirtuin family (Sirt1-7), out of which Sirt1, Sirt3, Sirt6, and Sirt7 exerted a positive effect on IPF (42). Overexpressions of Sirt inhibit the oxidative stress, pro-inflammatory cytokines IL-1β and p21 expressions, TGF-β1/Smad3 signaling pathway and mitochondrial DNA damage, elucidating a potential therapeutic approach to IPF. Oppositely, Talakatta observed an EMT and up-regulation of N-cadherin, Sirt3, and Sirt7 levels in diabetic cells exposed to high concentration of glucose (43), indicating that more studies should be carried out to demonstrate the exact role of Sirt on diabetic pulmonary fibrosis.

### Pro-Inflammatory and Pro-Fibrotic Factors

Connective tissue growth factor (CTGF) is a downstream mediator of the profibrotic properties of TGF-β, playing an important role in tissue remodeling and fibrosis. Up-regulated expression of CTGF is observed in several fibrotic organs such as kidney, heart, liver, skin, and lung (44). It was reported an increased expression level of CTGF in the lung tissues by the STZ induced type 1-like diabetic rat models, thereby indicating the adverse influence of diabetes on pulmonary fibrosis (8, 45). Besides, fibronectin, angiotensin II (Ang II), plasminogen activator inhibitor-1 (PAI-1) and other pro-inflammatory or pro-fibrotic factors were also found increased in diabetic mouse lungs, and they widely participated in the process of diabetic lung fibrosis by a variety of mechanisms (8, 46).

### Endoplasmic Reticulum Stress (ERS)

The endoplasmic reticulum (ER) is a kind of multi-functional organelle which is essential for the cellular homeostasis. Excessive demand on the ER or conditions could destruct the function of ER may cause the accumulation of unfolded or misfolded proteins, triggering ERS and activation of the unfolded protein response (UPR) (47). It has been demonstrated that ERS related markers in the lung were activated by bleomycin both *in vitro* and *in vivo* and treatment with ERS inhibitors resulted in the reduction of fibroblast proliferation and improvement of lung functions, suggesting a potential role of ERS for the pathogenesis of IPF (48, 49). Also, ERS is found associated with the β cell failure in type 1 and type 2 diabetes (47, 50). However, the role of ERS in diabetic pulmonary fibrosis is still unclear. Whether hyperglycemia could act as a stimulus inducing ERS in the process of diabetic pulmonary fibrosis or not remains to be further studied.

To conclude, activation of multiple pathways in the high-glucose environment results in abnormal intracellular stress and cytokine expressions, consequently causing loss of basal laminae integrity in alveolar-capillary barrier, failure of reepithelialization and reendothelialization, and epithelial-mesenchymal transition, and eventually, leading to destroyed lung architecture and pathological pulmonary fibrosis.

## Potential Application of Anti-Diabetic Drugs in IPF

### Metformin

At present, the therapeutic strategies and effects of IPF remain limited, although several tyrosine kinase inhibitors such as pirfenidone and nintedanib have come into our attention in recent years. Metformin is a classic oral anti-diabetic agent, which has been demonstrated anti-inflammatory, anti-angiogenetic and anti-fibrotic properties in animal models as well. In experimental murine models, studies support the role of metformin to reverse the established pulmonary fibrosis by facilitating deactivation and apoptosis of myofibroblasts via activating AMPK and ameliorating TGF-β signaling pathways (51–53). However, the pharmacokinetic data of oral metformin suggest that much of the concentration is in the gut, even after absorption. Besides, there is evidence suggesting that high dose of intravenous metformin has disconcerting effects on rats and inhibiting the viability of blood-brain barrier cell lines (54, 55). Thus, a pinpoint delivery of metformin to the lung such as inhalational drug delivery seems to be an attractive idea in future experimental studies.

### Glucagon Like Peptide-1 (GLP-1) Receptor Agonists

GLP-1 is an essential hormone for the regulation of insulin secretion, carbohydrate metabolism and appetite. GLP-1 receptor, a G protein coupled receptor, predominantly localized in β-cells of pancreas and smooth muscle cells of arteries and arterioles in kidney and lung, is mainly used as the target for anti-diabetic drugs (56). In recent years, several studies also found the anti-pulmonary fibrotic effect of GLP-1 receptor agonist in animals. Gou et al. demonstrated that liraglutide treatment significantly alleviated bleomycin (BLM)-induced lung damage and fibrosis in mice through inactivation of nuclear factor kappa-B (NF-κB) (57). Similarly, Oztay et al. found exendin-4 ameliorating hyperglycemia-mediated lung injury by reducing oxidative stress and stimulating cell proliferation (58).

### Peroxisome Proliferator-Activated Receptor-Gamma (PPAR-γ) Agonists

PPAR-γ belongs to nuclear hormone receptor superfamily, with action in many activities including alterations of metabolic and inflammatory responses. Recent studies also found an efficacy of PPAR-γ agonists in BLM-induced pulmonary fibrosis (59, 60). Rosiglitazone and ciglitazone were found inhibiting the fibrotic changes in TGF-β1-mediated EMT of alveolar epithelial cells, differentiation of myofbroblasts, and production of collagen in murine models, suggesting a therapeutic role of PPAR-γ ligands for fibrotic pulmonary disease (61, 62).

## Summary

Currently, incidences of diabetes and idiopathic pulmonary fibrosis are rising year by year, causing a heavy burden on patients. Objective evidence has found a link between the two diseases. Relying on limited research, we speculate that the sustained hyperglycemia in the body promotes the development of pulmonary fibrosis, through either directly damaging the alveolar epithelial cells (AECs) or participating in the generation of other pro-inflammatory and pro-fibrotic factors. Although the lung is not the main organ of diabetic complications, patients suffering from both diseases are reported a worsen prognosis. Increasing number of studies have found an anti-fibrotic effect of some anti-diabetic agents, and more attention and deeper studies on mechanism and intervention are needed for diabetic pulmonary fibrosis as well as an integrated follow-up system.

## Author Contributions

DW, YM, XT, YZ, and HF collaboratively conceptualized this manuscript. DW wrote the first draft of this manuscript. YM and XT wrote sections of the manuscript. All authors contributed to manuscript revision, read and approved the submitted version.

## Conflict of Interest

The authors declare that the research was conducted in the absence of any commercial or financial relationships that could be construed as a potential conflict of interest.

## Abbreviations

IPF: idiopathic pulmonary fibrosis
UIP: usual interstitial pneumonia
DM: diabetes mellitus
FVC: forced vital capacity
FEV_1_: forced expiratory volume in 1 s
VC: vital capacity
PEF: peak expiratory flow
DL_CO_: diffusing capacity of the lung for carbon monoxide
HR: hazard ratio
CI: confidence interval
CT: computed tomography
HRCT: high resolution computed tomography
BL: basal lamina
ECM: extracellular matrix
EMT: epithelial-mesenchymal transition
α-SMA: α-smooth muscle actin
AGEs: advanced glycation end-products
RAGEs: receptor for advanced glycation end-products
STZ: streptozotocin
ERK: extracellular regulated protein kinase
JNK: c-Jun N-terminal kinase
TGF-β: transforming growth factor beta
mRNA: messenger RNA
Sirt: sirtuins
NAD: nicotinamide adenine dinucleotide
CTGF: connective tissue growth factor
Ang-II: angiotensin II
PAI-1: plasminogen activator inhibitor-1
ERS: endoplasmic reticulum stress
ER: endoplasmic reticulum
UPR: unfolded protein response
AMPK: adenosine 5‘-monophosphate (AMP)-activated protein kinase
GLP-1: glucagon like peptide-1
BLM: bleomycin
NF-κB: nuclear factor kappa-B
PPAR-γ: peroxisome proliferator-activated receptor-gamma
AECs: alveolar epithelial cell.
